# B-1 cells in immunotoxicology: Mechanisms underlying their response to chemicals and particles

**DOI:** 10.3389/ftox.2023.960861

**Published:** 2023-04-18

**Authors:** Léa Hiéronimus, François Huaux

**Affiliations:** Louvain Centre for Toxicology and Applied Pharmacology (LTAP), Institut de Recherche Experimentale et Clinique (IREC), Université Catholique de Louvain (UCLouvain), Brussels, Belgium

**Keywords:** autoimmunity, autoantibody, granuloma, inflammation, B-1 lymphocytes, B1 cells

## Abstract

Since their discovery nearly 40 years ago, B-1 cells have continued to challenge the boundaries between innate and adaptive immunity, as well as myeloid and lymphoid functions. This B-cell subset ensures early immunity in neonates before the development of conventional B (B-2) cells and respond to immune injuries throughout life. B-1 cells are multifaceted and serve as natural- and induced-antibody-producing cells, phagocytic cells, antigen-presenting cells, and anti-/pro-inflammatory cytokine-releasing cells. This review retraces the origin of B-1 cells and their different roles in homeostatic and infectious conditions before focusing on pollutants comprising contact-sensitivity-inducing chemicals, endocrine disruptors, aryl hydrocarbon receptor (AHR) ligands, and reactive particles.

## 1 Introduction

B lymphocytes are white blood cells produced in the bone marrow and circulate in the blood and lymphatic vessels to defend the body against pathogens by secreting antibodies. B lymphocytes are classified according to their functions and differentiation. B-cell subsets mainly comprise B-2 cells, also called “conventional B cells” or “follicular B cells”. B-2 cells have been implicated in adaptive immunity and generate high-affinity antibodies with precise antigen specificity (LeBien and Tedder, 2008). The activation of B-2 cells results from an immune insult (e.g., an infection) sensed by immune cells, such as T and dendritic cells. Cross-talk between these cells is necessary for optimal adaptive humoral immune responses (den Haan et al., 2014). Plasma cells are fully differentiated B cells dedicated to releasing large amounts of antibodies of one isotype, and memory cells are long-lived B cells that provide long-term immunity (Cancro and Tomayko, 2021).

Less well-described, B-cell subsets also comprise innate-related cells. Indeed, antibody production by marginal zone (MZ) B and B-1 cells is T-independent. MZ B cells are localized in the secondary lymphoid organs and provide antibodies against circulating blood-borne pathogens (Martin et al., 2001). B-1 cells reside primarily in the mesothelial (peritoneal and pleural) cavities, representing the majority (up to 80%) of the B cells (Marcos et al., 1989; Kroese et al., 1992; Ghosn et al., 2010; Hiéronimus et al., 2021). Finally, among innate B cells, regulatory B cells (B reg) are those that reduce the level of inflammation and act as immunosuppressive cells (Dasgupta et al., 2020). Notably, B10 cells are a subset of B reg whose immunoregulatory effects are fully attributable to interleukin-10 production (Tedder, 2015).

This study aimed to highlight the wide range of B-1 cell responses. They remain one of the less-known subsets of B cells because they possess many facets and participate in various immune responses. Many of these functions are possibly undiscovered. In the present paper, we decided against using the term “B-1 lymphocytes” to refer to these cells as it limits them as lymphocytes. However, B-1 cells serve both lymphoid and myeloid functions as they express both sets of surface markers (Popi et al., 2009a). B-1 cells can be seen as pleiotropic multifaceted precursors or as a collection of several subsets of fully differentiated cells (Popi et al., 2012), including adaptive immune cells (Cole et al., 2009; Smith and Baumgarth, 2019). The new understanding that B-1 cells comprise a variety of subsets explains the exponentially growing interest toward these cells in applied immunology and toxicology (Haro et al., 2019; Aziz et al., 2020; Halperin et al., 2022; She et al., 2022).

In this review, we describe the developmental origin(s) of B-1 cells. After reporting their homeostatic functions under healthy conditions, we explain how pathogens unleash B-1 cells from their inhibitory environment and lead to their activation. We then highlight their unexpected roles (beneficial or deleterious) in immune responses to contact sensitivity-inducing chemicals, endocrine disruptors, aryl hydrocarbon receptor (AHR) ligands, and reactive particles.

## 2 The development of B-1 cells

### 2.1 The two existing models

**FIGURE 1:**
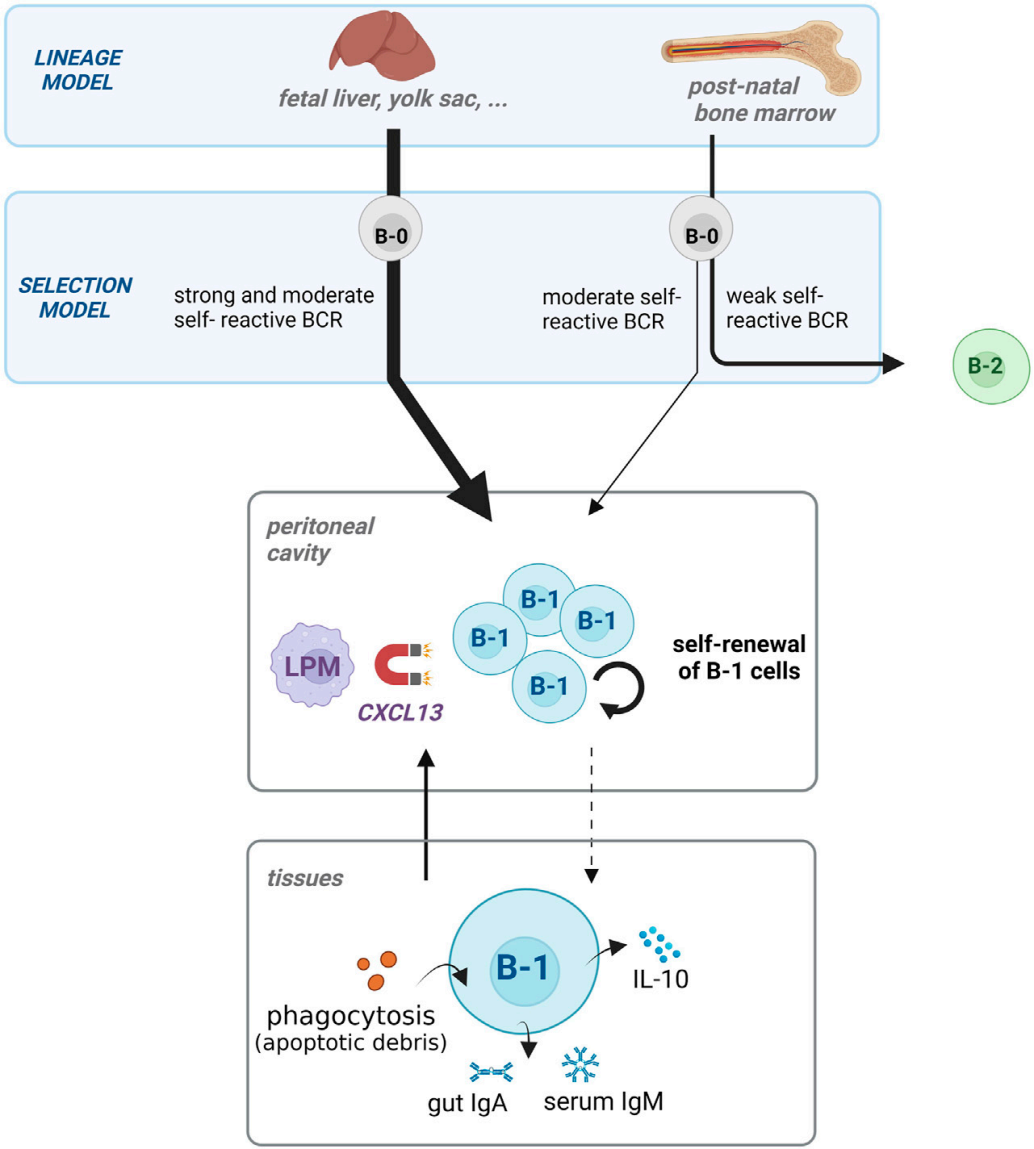
The development and function of B-1 cell pool during homeostasis. The development of B-1 cells occurs mostly in the fetus (lineage model). The bone marrow contributes only to a small addition of the B-1 cell pool in adults in contrast to B2 lymphocytes. The precursors (B-0) becoming B-1 cells have BCR with moderate or strong reactivity to self-antigens (selection model). B-1 cells are homed in the peritoneal cavity by CXCL13-releasing large peritoneal macrophages (LPM), where they self-renew. The three main functions of B-1 cells are apoptotic cell phagocytosis, IgM and IgA natural antibody production, and regulatory cytokine (IL-10) release, and contribute to maintaining homeostasis in the tissues.

The precise ontogeny of B-1 cells is still a matter of debate. Two different models regarding the B-1 cell origin exist. First, the “lineage model” postulates that the development into B-1 or B-2 cells predominantly depends on their respective precursors: fetal neonate for B-1 cells or post-natal bone marrow for B-2 cells. In addition to this concept, the authors also proposed an alternative “selection model,” which suggests a B cell receptor (BCR)-related origin. These two models are presented in Figure 1.

B-1 and B-2 cells originate from precursors physically and chronologically distant in the lineage model. Indeed, B-1 cells are mainly produced during early life in the yolk sac, placenta, peritoneum and neonatal liver (Baumgarth, 2017; Luo et al., 2022). They are generated in waves from different sequential early precursors, which do not persist after birth (Montecino-Rodriguez and Dorshkind, 2012; Baumgarth, 2017). Self-renewal, not bone marrow, maintains the pool of B-1 cells throughout life (Murakami et al., 1995; Sawai et al., 2016). B-1 cells proliferate autonomously under homeostatic conditions, mainly via IgM-autocrine loops (Krop et al., 1996; Nguyen et al., 2015; Kobayashi et al., 2020). Although new and more precise experiments revealed rare B-1 cell precursors in the post-natal bone marrow (Esplin et al., 2009; Ghosn et al., 2012), they do not significantly contribute to the pool of B-1 cells in adults (Luo et al., 2022). Nevertheless, while this model is mostly right, this discovery brings more nuance to the lineage model.

In the alternative “selection model,” B-1 and B-2 cells share a common B-cell precursor (B-0). B-0 becomes one or the other, according to the BCR on its surface. This model was supported by Graf et al. (2019) who used an inducible transgenic system to swap the BCR of mature B-2 and B-1 cells. Their study demonstrated that expressing a B-1 cell BCR is sufficient to efficiently transform a mature B-2 cell into a B-1 cell. Indeed, B-1 cell-BCRs are very similar (Kantor et al., 1997; Vale et al., 2015) and most B-1 cells target similar self-antigens (Tsuji et al., 2020). Thus, strong and intermediate BCR-signaling (i.e., via self-antigen recognition) on B-0 leads to the development of B-1 cells (Hardy, 2006; Ghosh et al., 2022). While B-1 cells undergo this “positive selection,” B-2 cells follow the opposite trend and are derived from B-0s which are weakly self-reactive.

These two models were initially thought to be mutually exclusive but tend to converge based on new findings. The idea of “layered immunity” indicates that different rules controlling the immunity apply at different ages (Tornberg and Holmberg, 1995; Budeus et al., 2021). This concept comes from the different constraints faced before and after birth. B-2 cells are generated throughout life to produce antibodies that recognize a vast diversity of antigens, notably to fight infectious agents. These features are not shared by B-1 cells, whose antibodies often target the same self-antigens. The new notion of “window of opportunities” explains that the unique origin of B-1 cells, in different waves in the early life, confers them relaxed rules of immune tolerance, including positive selection in response to self-antigens (Hardy and Hayakawa, 2015; Wong et al., 2019). The B-1 cell-reactivities that emerge during this window are still somehow regulated. Indeed, there is a remarkable similarity of the BCR-segments used by B-1 cells (Rowley et al., 2007), amplified by the fetal absence of the enzyme necessary to diversify the BCR (Kantor et al., 1997).

### 2.2 Mechanisms inhibiting B-1 cell activation

B-1 cells are reactive to self-antigens and can be activated independently of other cells, as opposed to the T cell-dependent activation of B-2 cells (Martin et al., 2001; Alugupalli et al., 2004). Thus, B-1 cells require inhibitory mechanisms to regulate their spontaneous activation. Their localization plays a major role in their response and is regulated by macrophages (Figure 1). The peritoneum acts as a reservoir and an inhibitory environment for B-1 cells (Kawahara et al., 2003; Sindhava et al., 2010; Choi et al., 2012). Large peritoneal macrophages (LPMs) release abundant CXCL13, which is crucial for the localization of B-1 cells (Ansel et al., 2002). The LPMs retain B-1 cells via CXCL-13-mediated homing, as this cavity is closed from the circulation (Okabe and Medzhitov, 2014). Thus, retaining them in the mesothelial cavities decreases the likelihood of B-1 cells encountering activating signals.

Other mechanisms have also been implicated in controlling B-1 cell activation and antibody release. To produce antibodies, B-1 cells require simultaneous toll-like receptor (TLR) and BCR signaling (Sindhava et al., 2010; Sindhava and Bondada, 2012; Alhakeem et al., 2015). Only certain TLR signaling pathways collaborate with precise B-1-cell BCRs (Genestier et al., 2007; Savage et al., 2019). The binding of nucleic acids to endosomal TLR and phospholipids to BCRs demonstrate a pair of TLR and BCR signals that synergize. These mediators are both present under homeostatic conditions during cell death and mediate B-1 cell-produced anti-phospholipid antibodies in the steady state (Kreuk et al., 2019). Thus, B-1 cell-related antibody production is triggered by innate signals and is not strictly dependent on pathogens. In germ-free mice, most natural antibodies are produced by the spleen- and bone marrow-resident B-1 cells (Choi et al., 2012). These microenvironments contain a low number and frequency of B-1 cells, whose activation is notably regulated via CXCR4+ Tregs (Stoermann et al., 2007; Elias et al., 2022). Similar to T cells, B-1 cells express the inhibitory surface markers CD5 (Berland and Wortisoorelbeke, 2002; Smith and Baumgarth, 2019), PD-1 (Blevins et al., 2021; Zhou et al., 2022), and CTLA-4 (Yang et al., 2021) to regulate their activation.

## 3 B-1 cells in healthy conditions

B-1 cells perform multiple housekeeping functions during the steady state. They are responsible for the efficient clearance and immune tolerance of cellular debris during fetal life and in adults (Parra et al., 2012; Miles et al., 2018). Indeed, B-1 cells release T-independent antibodies targeting apoptotic cells, phagocytose debris, and release anti-inflammatory cytokines (Ogden et al., 2005). The B1 functions during steady state described in the text below are also illustrated at the bottom of the Figure 1.

### 3.1 Natural antibodies

B-1 cells are already present and are active before the development of adaptive immunity. They produce natural antibodies with low affinity and poly-specificity (recognizing multiple antigens), which serve as a ready-made arsenal to protect the newborn (Baumgarth et al., 2005; Ordoñez et al., 2018). The main isotype of natural antibodies is immunoglobulin M (IgM). The vast majority (>80%) of natural serum IgM is produced by B-1 cells (Choi and Baumgarth, 2008; Choi et al., 2012). B-1 cells are also capable of class switching to produce IgA (up to 50% of physiological serum IgA) and other isotypes (Baumgarth et al., 1999; Baumgarth et al., 2005). B-1 cells in the intestinal lamina propria (Okabe and Medzhitov, 2014; New et al., 2020) represent up to half of the physiological gut IgA-producing cells (Kroese et al., 1989).

Another characteristic of B-1 cell-produced antibodies is their preferential targeting of non-protein antigens, such as lipids (i.e., cardiolipin, phosphorylcholine (PC), oxidized low-density lipoprotein (oxLDL) (Shaw et al., 2000; Ordoñez et al., 2018) and nucleotides (Enghard et al., 2010). Additionally, these are “altered-” or “neo-” self-antigens. Although intracellular in healthy conditions, they change conformation and become available on “unwanted” cells, including apoptotic cells. Altered self-antigens function as tags to facilitate clearance of immunogenic cells and cellular debris (Navratil et al., 2004; Eggleton et al., 2008). For example, B-1 cells are the main producers of natural anti-PC antibodies (Mercolino et al., 1988; Masmoudi et al., 1990). This altered self-antigen becomes exposed during cell death, notably on the membrane of lysed-red blood cells (RBC) (Cox and Hardy, 1985). Natural IgM specific for altered self-antigen recognize cells during early apoptosis (Mercolino et al., 1988; Kim, 2010) and promote local deposition of “eat-me signals” (C1q and mannose-binding lectin) for their rapid clearance (Ogden et al., 2005; Chen et al., 2009).

### 3.2 Phagocytosis

B-1 cells share several characteristics with macrophages. Both cell types express myeloid cell markers, such as F4/80, class II major histocompatibility complex molecules (MHC II), and CD80/CD86 (Popi et al., 2009a; Parra et al., 2012; Popi, 2015). The phagocytic ability of B-1 cells is greater than that of any other B cells. While B-2 cells can uptake pathogens via internalization of the antigen-BCR complex, B-1 cells are professional phagocytes (Gao et al., 2012) competent to engulf large bodies (>0.5 µm), including apoptotic debris through actin polymerization (Brito et al., 2010; Parra et al., 2012).

### 3.3 Interleukin-10 production

B-1 cells are the main source of B-cell-produced interleukin-10 (IL-10) in homeostatic conditions (O'Garra et al., 1992). The spontaneous release of IL-10 by peritoneal B-1 cells is not shared by splenic B-2 cells and has thus been extensively studied. Unlike B-2 cells, separate TLR or BCR signaling promotes IL-10 release (Sindhava et al., 2010; Sindhava and Bondada, 2012; Alhakeem et al., 2015). IL-10 signaling prevents B-1 cell activation, blocks the cell cycle, and prevents IgM release (Alhakeem et al., 2015; O'Garra et al., 1990). Thus, the spontaneous release of IL-10 maintains peritoneal B-1 cells in a state of slow proliferation and less IgM release. In addition to autocrine effects, IL-10 released from B-1 cells stimulates other cells to release IL-10. This cytokine helps maintaining tolerance, notably during physiologically occurring apoptosis (Miles et al., 2018).

## 4 B-1 cells in pathological conditions

**FIGURE 2:**
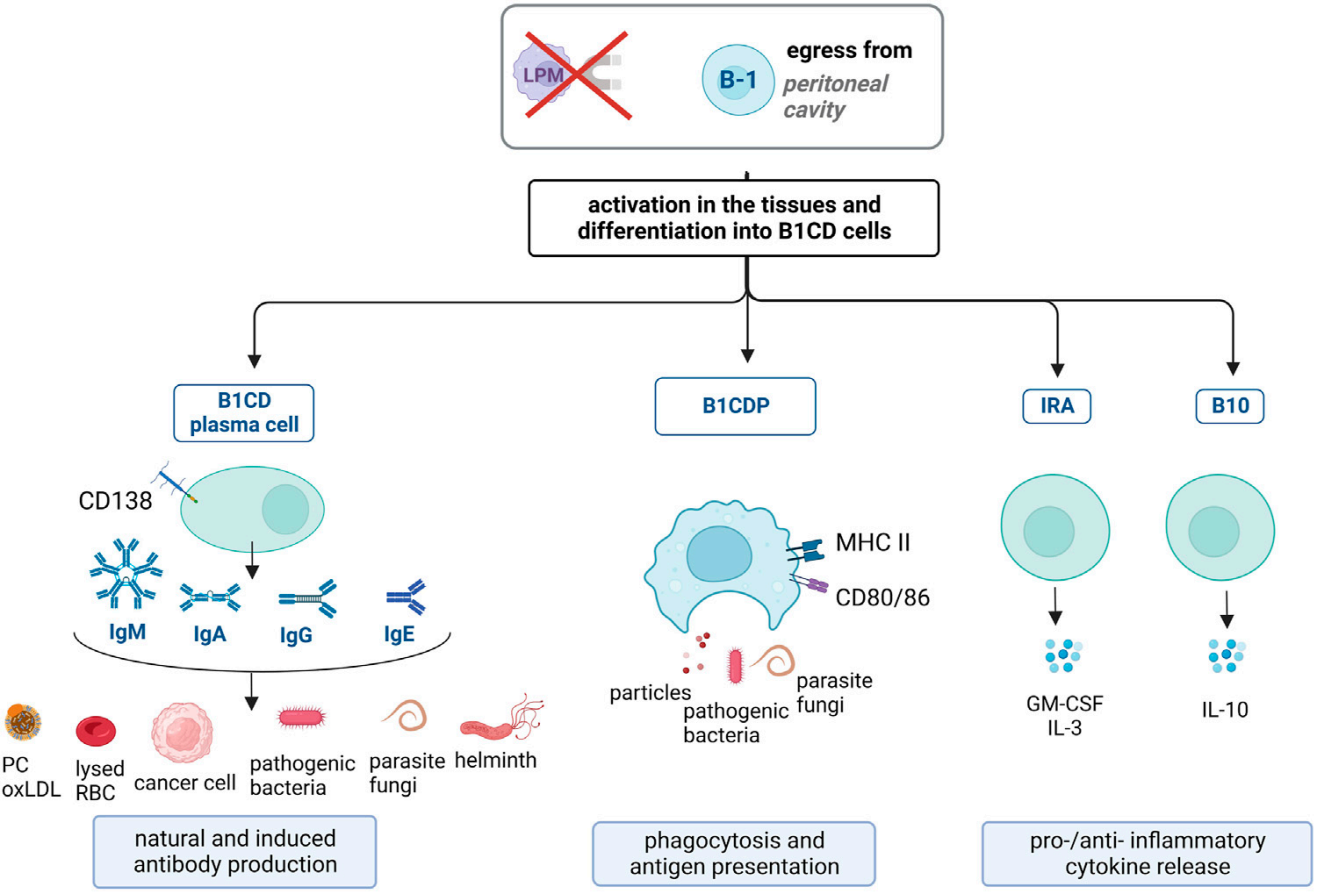
B-1 cell responses in pathological conditions. Under pathological conditions, B-1 cells leave mesothelial cavities and get activated. They differentiate into B-1 cell-derived (B1CD) cells, comprising four subpopulations. B1CD plasma cells are CD138^+^ and release natural antibodies (mostly IgM, with low specificity to (self-)antigens, including phosphorylcholine (PC) and oxLDL present on apoptotic cells, lysed red blood cells (RBC) and cancer cells) and induced antibodies (IgM, IgA, IgG, and IgE, with high specificity to antigens, including pathogens). B1CD phagocytes (B1CDP) are phenotypically similar to macrophages, phagocytose and present antigens to T cells via MHC II and CD80/CD86. Innate response activator (IRA) release pro-inflammatory GM-CSF and IL-3. B10 cells exclusively release immunosuppressive IL-10.

In response to infection or inflammation, B-1 cells from the mesothelial cavities become activated (and often gain surface CD11b expression) (Ghosn et al., 2008) and migrate to the injury site and lymphoid organs. A fraction of the migrated B-1 cells remains undifferentiated and continues their three synergistic functions (natural antibody production, phagocytosis and IL-10 release) (Rauch et al., 2012). The remaining B-1 cells differentiate into different B-1 cell-derived cells (B1CD) (Lopes and Mariano, 2009). Differentiation signals have not yet been clearly determined. However, it has been established that the pool of B-1 cells adapts to the pathogen under inflammatory conditions (Patel and Kearney, 2015; New et al., 2020). Thus, whether studied at a given time or over a lifetime, the B-1 cell subset contains cells with variations in their functions/specificities, as explained below and in Figure 2 (Halperin et al., 2022).

### 4.1 Natural and induced antibody-producing B1CD cells

Natural antibodies produced by B-1 cells under homeostatic conditions are beneficial for fighting infection. While natural antibodies target altered-self antigens on apoptotic cells, they also help clear pathogens (Ordoñez et al., 2018; Webster et al., 2022) and tumor antigens (Díaz-Zaragoza et al., 2015; Haro et al., 2019). Indeed, natural antibodies are non-specific and possess low affinity for their antigens. Hence, these poly-specific antibodies take advantage of the resemblance of epitopes, such as PC on both damaged cells and pathogens (Patel and Kearney, 2015). B-1 cells ensure first-line defense because their natural antibodies are already present in the blood and quickly bind to pathogens during early infection (Haas et al., 2005; Smith and Baumgarth, 2019). During infection, the levels of natural antibodies remain stable in the serum, as they are produced independently of pathogens (Baumgarth et al., 1999). However, some B-1 cells quickly migrate from the peritoneal cavity to the infected zone and secondary lymphoid organs (Choi and Baumgarth, 2008; Russo and Mariano, 2010). The lack of T-cell dependency allows B-1 cells to rapidly release high levels of natural antibodies locally (Baumgarth et al., 1999; Ordoñez et al., 2018). Some B-1 cells differentiate into CD138+ B-1 cell-derived plasma cells (B1CD plasma cells) to release even higher amounts of (poly-specific) natural IgM (Yang et al., 2007; Choi and Baumgarth, 2008; Holodick et al., 2014; Savage et al., 2017; Ordoñez et al., 2018). Natural anti-PC antibodies, produced by B-1 cells and B1CD plasma cells, have been well characterized and shown to be protective against the development of infections with helminths (Ubeira et al., 1987), bacteria (such as *Streptococcus pneumoniae* (Briles et al., 1982; Haas et al., 2005) and mycobacteria (Ordoñez et al., 2018).

Moreover, B1CD plasma cells also release “induced antibodies” or “pathogen-specific antibodies” in response to infection. A fraction of migrated B-1 cells undergo affinity maturation, increasing their specificity to the antigen (Chen et al., 2003; Alugupalli et al., 2004) and class-switch toward IgA (Kroese et al., 1989; New et al., 2020), IgG (Hirose et al., 1993; Chen et al., 2003) and IgE (Oliveira et al., 2005; Ghosh et al., 2012). These induced antibodies represent new additions to the B-1 cell repertoire. Antigen-specific B1CD plasma cells act as memory B-1 cells (Choi and Baumgarth, 2008; Smith and Baumgarth, 2019) and provide long-term protection (Cole et al., 2009). After the resolution of the activating signal, B1CD plasma and B-1 cells migrate back into the body cavities via CXCL13-mediated homing (Ansel et al., 2002; Ghosn et al., 2008). Repeated or prolonged activation of B-1 cells and migration out of the peritoneal cavity lead to expansion, affinity maturation or class-switching to IgG (Murakami et al., 1997; Ishikawa and Matsushima, 2007; Enghard et al., 2010). In older individuals, B-1 cells are less likely to spontaneously release low-affinity natural autoIgM because a fraction of them have adapted to pathogens and now release high-affinity IgG antibodies (Holodick et al., 2016; Rodriguez-Zhurbenko et al., 2019; Luo et al., 2022). It seems that B-1 cells also develop adaptive immune responses to cell death and apoptosis. In some cases, B-1 cells release harmful antibodies against lysed red blood cells (RBCs) that no longer clear apoptotic debris but instead destroy healthy RBC, leading to autoimmune hemolytic anemia (Murakami et al., 1992; Murakami et al., 1995; Murakami and Honjo, 1996; Dei Zotti et al., 2021). These pathological autoantibodies can activate the complement pathway, Fc receptor-mediated phagocytosis, or agglutination of healthy RBCs (Stahl et al., 2000).

### 4.2 B-1 cell-derived phagocytes (B1CDP)

Although B-1 cells phagocytose apoptotic cells and pathogens (Brito et al., 2010; Gao et al., 2012), their phagocytic ability is even stronger after differentiation into B1CDP (Figure 2). The uptake of pathogens by B-1 cells can be antigen-independent, similar to macrophages (Parra et al., 2012), or antigen-dependent. The latter is preferentially used by B-1 cells (Cruz-Leal et al., 2014; Vo et al., 2014) and serves during antigen presentation and differentiation into B1CDP (Popi et al., 2016). B-1 cells then use two different receptors to bind and phagocytose pathogens. Antigen recognition via BCR, when combined with TLR signaling, leads to internalization of the antigen–BCR complex and phagocytosis of the pathogen (Mussalem et al., 2012; Parra et al., 2012). Antigen peptides are then processed in the phagolysosome to be presented via MHC II (see below) (Gao et al., 2012; Vo et al., 2014; Yang et al., 2021).

While B-1 cells using this type of phagocytosis transiently lose IgM-BCR surface expression during internalization (Yang et al., 2021), some B-1 cells proceed further and progressively downregulate B cell-associated genes (Popi et al., 2009a). Besides the low expression of CD19 and IgM, they become morphologically and phenotypically similar to monocyte-derived macrophages (Almeida et al., 2001; Popi et al., 2012). Like in macrophages, B1CDP phagocytosis becomes predominantly antigen-independent (Vo et al., 2014). B1CDP migrate from the peritoneum to the infection/inflammation site, where they ingest apoptotic debris, particles, parasites, and bacteria for their rapid clearance (Almeida et al., 2001; Popi et al., 2009b; Popi et al., 2012; Arcanjo et al., 2015).

In response to persistent foreign bodies, macrophages form compact cellular aggregates and granulomas to isolate foreign bodies from the surrounding tissues (Pagan and Ramakrishnan, 2018). In addition to macrophages, B-1 cells are also crucial for granuloma formation. After *mycobacterium* infection, B-1 cells migrate to the infected lungs to promote compact granuloma formation, whereas the absence of B-1 cells (Xid mouse model) leads to diffused, rather than localized, lung lesions (Russo and Mariano, 2010). B-1 cells collaborate with macrophages to drive granuloma formation, as illustrated using an *in vitro* model of *Paracoccidioides brasiliensis* exposure (Vigna et al., 2006). Further demonstrating their similar phenotype and functions, B-1 cells migrate to the foreign body and can be found inside granulomas, then become B1CDP and fuse with macrophages to form plurinucleated giant cells (Almeida et al., 2001). By sharing their cytoplasm, fused cells create longer pseudopodia and phagocytose larger foreign bodies (Milde et al., 2015).

### 4.3 Antigen-presenting functions of B1CDP and B-1 cells

T cell activation requires two synergizing signals: one from the ligation of the T cell receptor (TCR) with the antigen-bearing MHC and the second from the ligation of T cell-costimulatory molecules, such as CD28 with either CD80 (B7-1) or CD86 (B7-2). Dendritic cells usually serve as MHC and CD80/CD86+ antigen-presenting cells (den Haan et al., 2014). Compared to B-2 cells, B-1 cells express high levels of all these costimulatory molecules (CD80, CD86, CD24, LFA-1, and ICAM-1) as well as MHC II (Mohan et al., 1998; Vigna et al., 2002; Parra et al., 2012) (Figure 2). This expression is constitutive and increases after exposure to pathogens, notably while B-1 cells differentiate into B1CDP (Mussalem et al., 2012).

B-1 cells in the peritoneum and lymphoid organs have high antigen presentation activity to T cells (Mohan et al., 1998; Parra et al., 2012). It has been reported that B-1 cells mediate the differentiation of most T cells, including Th17, Th1, Treg, Th2 and PD-1+ T cells (Zhong et al., 2007; Margry et al., 2014; Enyindah-Asonye et al., 2019). The signals that determine the direction of T cell-differentiation are not yet known. The egress of MHC II-positive B-1 cells outside the peritoneal inhibitory environment in the T cell-enriched thymus and spleen is suspected to be implicated in the activation of autoreactive T cells, leading to excessive inflammation and autoantibody production (Ishikawa and Matsushima, 2007; Pfau et al., 2014a; Pfau et al., 2014b). In their review on the antigen-presenting ability of B-1 cells, Popi et al. mentioned that B-1 cells prime virgin T cells, while B-2 cells re-activate memory T cells (Popi et al., 2016).

### 4.4 Innate response activator (IRA) cells and B-1 cell-derived B10 cells

After lipopolysaccharide (LPS) injection, B-1 cells from mesothelial cavities migrate to the injection site and secondary lymphoid organs, and some differentiate into IRA cells. Unlike B-1 cells, IRA cells release high amounts of granulocyte-macrophage colony-stimulating factor (GM-CSF) and IL-3 to activate innate immune cells (Rauch et al., 2012; Chousterman and Swirski, 2015) (Figure 2). They also maintain their own activation, as they are both the producers and targets of GM-CSF. This cytokine increases survival, proliferation, and differentiation of dendritic cells, monocytes, and macrophages, which in turn release pro-inflammatory cytokines and activate T cells and Th1 responses (Hilgendorf et al., 2014). IRA cells are implicated in inflammatory diseases, including multiple sclerosis (MS) (Li et al., 2015), sepsis-induced cytokine storms (Rauch et al., 2012), and atherosclerosis (Hilgendorf et al., 2014). In addition, IRA cells are CD138+ cells and release IgM. In the case of bacterial infection, IRA cells locally release opsonizing IgM, which is sufficient to confer survival to infected hosts (Rauch et al., 2012; Weber et al., 2014). IRA cells are also present in human tonsils and probably act as the first line of defense against upper respiratory tract infection (Chiappini et al., 2015).

B-1 cells exhibit immunosuppressive activity via antigen-presenting function and spontaneous IL-10 release (Margry et al., 2014; Dasgupta et al., 2020). B-1 cells differentiate into B10 cells (Matsushita et al., 2016; Ran et al., 2020) in response to innate signals that combine both TLR and BCR- or CD40-mediated activation (Yanaba et al., 2009; Dasgupta et al., 2020). B10 cells almost exclusively release IL-10 (Yanaba et al., 2009) This secretion impedes the concomitant release of IgM in B10 cells (Maseda et al., 2012; Tedder, 2015). In cases of infection or inflammation, B10 cells expand and migrate to the injury site or secondary lymphoid organs to dampen inflammation and promote Treg accumulation, as observed in experimental autoimmune encephalomyelitis (Matsushita et al., 2010), *Listeria monocytogene*s infection (Horikawa et al., 2013), allergy (Matsushita et al., 2016) and other pathologies [reviewed in (DiLillo et al., 2010)].

## 5 B-1 cell responses to reactive chemicals and particles

**FIGURE 3:**
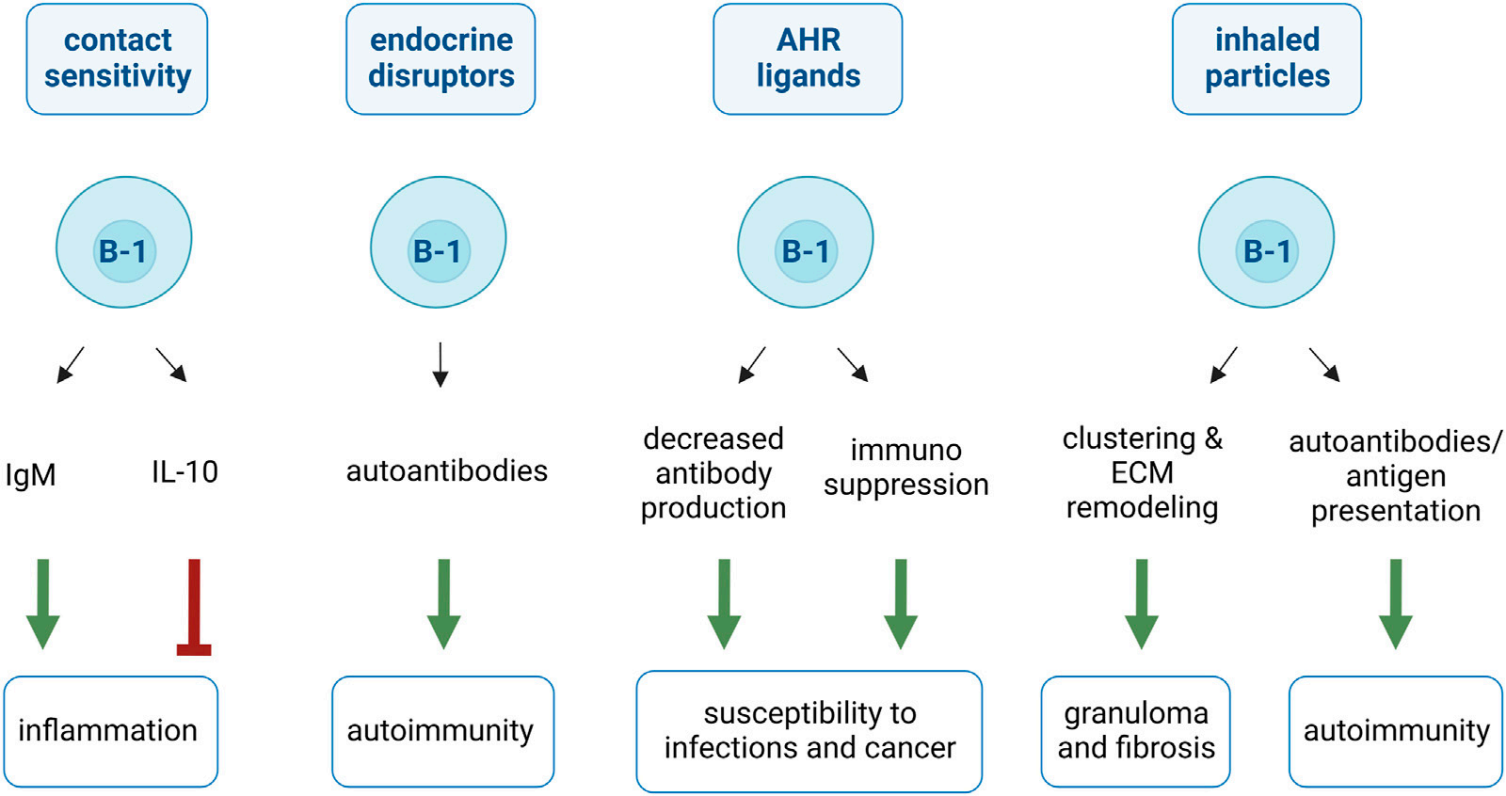
Proposed effects of B-1 cells in response to chemicals and particles. B-1 cells sense chemicals and particles and amplify (green arrows) or dampen (red arrow) associated diseases. B-1 cells are activated by contact sensitivity-inducing haptens, produce IgM and promote inflammatory pathology. B-1 cells also limit inflammatory responses by releasing anti-inflammatory cytokines (IL-10). B-1 cells promote autoimmunity by sensing endocrine disruptors and increasing their autoantibody production. AHR ligands reduce B-1 cell ability to secrete antibodies but favor their immunosuppressive activities. These two effects explain susceptibility to infection and cancer associated to AHR ligands. B-1 cells mediate inhaled particle-induced granuloma formation and fibrosis by clustering cells and particles and remodeling extracellular matrix (ECM). B-1 cells are also implicated in inhaled particle-induced autoimmune diseases by secreting autoantibodies and presenting self-antigens.

The implication of B-1 cells in pathogen-mediated diseases has been studied since their discovery. Thus, sufficient observations have allowed for the distinction between B-1 and B1CD cell responses to infections and inflammation. Understanding the implications of B-1 cells in response to chemical compounds and inhaled particles is still new in immunotoxicology and there is limited research regarding the responses of B-1 cells to pollutants. Nevertheless, we have highlighted this under-studied immune population and depicted their probable role in responses to reactive agents in the following text and Figure 3. Also, B-1 cells are often defined more by their function than by their panel of surface markers. Therefore, in the present section, all B-1 and B1CD cells are considered “B-1 cells”.

### 5.1 B-1 cells and allergic contact sensitivity (CS)

Contact sensitivity, also called skin allergy or allergic dermatitis, is a typical example of delayed-type hypersensitivity. It is mediated by effector T cells that induce an inflammatory reaction against exogenous or endogenous antigens. Other cells, such as monocytes, eosinophils, and neutrophils, are also involved (Vocanson et al., 2009). The team of Askenase found that B-1 cells are crucial in the development of CS in addition to T cells [reviewed in (Askenase, 2001)]. The pathology is attributable to B-1 cells and not to other types of B cells because the transfer of CS-B-1 cells is sufficient to induce CS in untreated mice (Itakura et al., 2005).

CS can be triggered by chemicals, such as trinitrophenyl chloride (TNP-Cl), oxazolone (OX), and fluorescein isothiocyanate (FITC) (Campos et al., 2006). After skin sensitization, some haptens are absorbed by the skin and bind, often covalently, skin proteins. The hapten-self protein conjugates constitute the neoantigens. Some of them are drained to the peritoneum (Askenase, 2001), where they encounter and activate B-1 cells (Itakura et al., 2005) alongside signals produced by activated NK T cells such as IL-5 (Itakura et al., 2013), IL-33 (Komai-Koma et al., 2011) or IL-4 (Askenase et al., 2005). Stimulated B-1 cells migrate to the spleen and lymph nodes via IL-4-related signaling and CXCL13 chemoattraction to produce IgM antibodies specific to the conjugates. This novel IgM circulates in the serum and leads to the recruitment of T cells in lymphoid organs within few hours (Mori et al., 2000; Askenase, 2001). Finally, T cells are activated in the spleen by dendritic cells, which are derived from Langerhans cells that have processed neoantigens in the skin. These T cells become CS-effector T cells and return to circulation 4 days after CS initiation (Askenase, 2001).

During the second encounter with the chemical, the induced IgM targeting anti-hapten/self-protein conjugate is already circulating and is thus rapidly found in the skin. IgM produced by B-1 cells activates the classical complement pathway and promotes recruitment of T cells, including CS-effector T cells, to the skin for induction of inflammation (Askenase et al., 1999; Tsuji et al., 2000; Askenase et al., 2015). Further demonstrating the crucial role of these B-1 cell-produced antibodies in the pathogenesis, injections with recombinant anti-hapten pentameric IgM trigger CS-symptoms in naïve mice (Paliwal et al., 2002) (Figure 3).

Alternatively, during the first exposure to a CS-inducing agent, some B-1 cells migrate to the spleen and differentiate into B10 cells. These cells release IL-10 to dampen T cell-mediated inflammatory responses and promote Treg expansion. The transfer of B-1 cell-derived B10 cells significantly attenuates CS responses (Yanaba et al., 2008; Matsushita et al., 2016). These contradictory findings strongly suggested that B-1 cells differentiate into deleterious antigen-specific IgM-releasing or beneficial B-1 cell-derived B10 cells (Figure 3).

### 5.2 B-1 cells and environmental estrogens

Estrogens enhance polyclonal B-cell activation and self-reactive B-cell survival (Fish, 2008). This is especially true for the B-1 cells. Estrogen receptors are highly expressed by the B-1 subset in older individuals compared to that in young B-1 and B-2 cells (Yurino et al., 2004; Diehl et al., 2011). Estrogens maintain the population of self-reactive B-1 cells, leading to an increased risk of autoantibody production in females, especially in older individuals (Figure 3) (Ansar Ahmed et al., 1989; Webster et al., 2022). Estrogen enhances the levels of anti-lysed RBC antibodies (Yurino et al., 2004). Interestingly, primary autoimmune hemolytic anemia, caused by pathological anti-RBC antibodies produced by B-1 cells, is most prevalent in aged and female individuals (Dei Zotti et al., 2021).

Endocrine disruptors (ED) or environmental estrogens found in plastics (bisphenol-A and phthalates), detergents, surfactants, pesticides and industrial chemicals (Tapiero et al., 2002) also activate B-1 cells. Schielen et al. (1995b) observed specific activation of B-1 cells in a rat model exposed to hexachlorobenzene (HCB). This ED is known to induce pathological skin lesions in human and rats (Michielsen et al., 1997; Michielsen et al., 1999). HCB treatment elevated the number of splenic IgM- and IgG-producing cells. Among B-cell subsets in the spleen of treated rats, only B-1 cells were expanded. The enhanced antibodies measured in the serum were similar to natural antibodies and characterized by their poly-reactivity to altered self-antigens including DNA and PC (Schielen et al., 1993; Schielen et al., 1995a). The significant correlation between IgM levels and skin lesion severity in HCB-treated animals strongly suggested a role of B-1 cells in the development of inflammatory lesions (Schielen et al., 1995b).

The impact of ED on B-1 cell-mediated autoimmunity has been evaluated in a lupus-prone model, NZB/NZW F1 mice. Progressive accumulation of splenic B-1 cells is observed in old NZB/NZW F1 mice. It leads to pathological anti-DNA IgG deposition, resulting in immune complex formation (Ito et al., 2004; Ishikawa and Matsushima, 2007). These antibodies originate from natural anti-DNA IgM produced by B-1 cells (Enghard et al., 2010). The class-switch from beneficial IgM-to pathological IgG antibodies reactive to DNA can be modulated by sex-hormones and is accelerated by estrogens (Roubinian et al., 1978). Yurino et al. (2004) reported the effects of ED bisphenol-A and diethylstilbestrol on B-1 cells in ovarectomized NZB/NZW F1 mice. ED worsened immune complex deposition in the kidneys and significantly increased the levels of anti-DNA IgG in addition to anti-RBC antibodies. The number of splenic B-1 cells were not further increased by ED in this lupus-prone model. *In vitro,* environmental estrogens stimulate the release of IgM, including anti-DNA IgM, by B-1 cells, especially in old mice. This is not the case of B-2 cells, which release very little IgM and are not activated by these molecules (Yurino et al., 2004). It remains to be elucidated whether Ig class switching from IgM to IgG occurs in ED-exposed B-1 cells.

### 5.3 B-1 cells and the AHR

The AHR is a crucial barrier organ sensor (reviewed in (Cella and Colonna, 2015; Gutiérrez-Vázquez and Quintana, 2018). AHR is expressed by both innate and adaptive immune cells (Wang et al., 2020) as well as by tissue-specific cells, such as epithelial cells (Qian et al., 2022) and keratinocytes (Uberoi et al., 2021). This transcription factor modulates immune responses and favors either immunosuppressive responses or Th1/Th17 inflammation (Quintana et al., 2008; Duarte et al., 2013). It is an evolutionarily old intracellular receptor (Hahn et al., 2017) that acts as a transcription factor and integrates signals from various sources. AHR ligands include dietary chemicals [indole-3-carbinol (Kahalehili et al., 2020), polyphenols (Gouédard et al., 2004; Mohammadi-Bardbori et al., 2012), glucosinolates (Koper et al., 2020)], endogenous small chemicals [tryptophan and metabolites (Rannug et al., 1995; Zelante et al., 2013)], and other pollutants [dioxins (Faiad et al., 2016; Miret et al., 2016), polychlorobiphenyls (Farmahin et al., 2016; Liu et al., 2019) benzo [a] pyrene (Nakatsuru et al., 2004), and diesel exhaust particles (Weng et al., 2018)].

Peritoneal CD5^+^ B-1 cells are among the B cells that express AHR in greater levels (Villa et al., 2017). The impact of AHR signaling in B-1 cells was studied in a CD5^+^ murine B-cell lymphoma line (CH12.LX), often considered a B-1 cell model. CH12.LX cells express surface CD11b, MHC II, alpha-4-integrin, IgM, and B220 and lack B-2 cell-associated CD23 (Rasmussen and Pfau, 2012). AHR is greatly expressed in CH12.LX (Sulentic et al., 1998). 2,3,7,8-tetrachlorodibenzo-p-dioxin (TCDD) prevents CH12.LX from releasing antibodies in response to LPS (Sulentic et al., 1998). Follow-up studies reviewed by Sher and Monti demonstrated that TCDD-mediated AHR signaling suppresses pathways necessary for plasma cell differentiation (Sherr and Monti, 2013). Thus, B-1 cells may respond to AHR agonists by limiting the antibody release (Figure 3).

Recent studies on human samples presented similar results on the importance of AHR in B-1-like cells (Blevins et al., 2021; Zhou et al., 2022). Human CD5^+^ B cells resemble mouse B-1 cells, with innate-like cells releasing the vast majority of the natural IgM in an age-related manner. CD5^+^ B cells express more basal AHR than CD5^−^ B cells, as well as upstream mediators of programmed death 1 and 2 (PD-1/-2) and its ligands (PDL-1 and PDL-2). Activation of PD-1 initiates signaling cascades that suppress cellular immune responses. It was also found that TCDD-mediated AHR activation on CD5^+^ B cells significantly increased surface PD-1 and inhibited IgM production. Additionally, lymphocyte-specific protein tyrosine kinase (LCK) is upregulated via AHR activation in these cells and further suppresses IgM release by acting on the same pathway (Blevins et al., 2021; Zhou et al., 2022). Interestingly, the increase in LCK has been implicated as a biomarker for the progression of chronic lymphoid leukemia. This cancer could originate from B-1 cells, as it is characterized by CD5^+^ B cell/macrophage bi-phenotype tumor cells (Majolini et al., 1998; Borrello and Phipps, 1999). These data suggest that B-1 cells may play a role in TCDD-induced immunosuppressive diseases, such as poor infection control and leukemia (Warren et al., 2000; Vorderstrasse et al., 2003; Gaspard et al., 2022) (Figure 3).

### 5.4 B-1 cells and particle-induced granuloma

B-1 cells have been implicated in the formation of granulomas triggered by inorganic foreign bodies. Granuloma-inducing particles [silica (Hiéronimus et al., 2021), carbon nanotubes (Hiéronimus et al., 2021) and asbestos (Pfau et al., 2014a; Pfau et al., 2014b)] specifically activate and lead to the migration of B-1 cells. They are recruited to and accumulate in the inflammatory site and lymphoid organ when granuloma formation is in progress. B-1 cells remain in these organs during particle-induced chronic responses (Pfau et al., 2014a; Pfau et al., 2014b; Hiéronimus et al., 2021). Mechanistically, a novel and peculiar property of B-1 cells has been demonstrated as they cluster granuloma-inducing particles. This property seems specific to B-1 cells and reactive particles, as it was not observed in B-2 cells or inert particles (Hiéronimus et al., 2021). Unlike macrophages, B-1 cells are resistant to the cytotoxicity of microparticles. This suggests that B-1 cells take advantage of their unique resistance to the toxicity of particles and cluster them, acting as a shield to protect the surrounding cells and avoid particle distribution in the tissue (Hiéronimus et al., 2021) (Figure 3).

B-1 cells also interact with their environment, cross-talk, and fuse with macrophages to form compact cellular aggregates in response to pathogens (Vigna et al., 2006; Russo and Mariano, 2010). This is also the case for particle-induced granuloma. Indeed, in response to silica particles, B cell-deficient mice (μMT-deficient mice) develop few cellular aggregates and deposit less collagen in their lungs, probably due to the loss of pro-fibrotic signals activating macrophages (Arras et al., 2006). B-1 cells collaborate with macrophages to form granuloma-like structures *in vitro*. Adding B-1 cells (and not B-2 cells) to macrophages increases the number and size of granuloma-like structures induced by carbon nanotubes (Hiéronimus et al., 2021). In addition, B-1 cells specifically stimulate macrophages to release tissue-inhibitor of metalloproteinase1 in fibrotic-granuloma-inducing conditions (Hiéronimus et al., 2021). Thus, B-1 cells promote both the formation and maturation of granulomas into fibrotic granulomas by acting on the macrophages (Figure 3).

### 5.5 B-1 cells and particle-induced autoimmunity

Occupational exposure to silica (Pollard, 2016; De Decker et al., 2018) and asbestos (Pfau et al., 2014a; Pfau et al., 2014b) is associated with a high risk of developing autoimmune diseases, such as systemic lupus erythematosus, rheumatoid arthritis, and systemic sclerosis. Considerable experimental data indicate that autoantibodies precede clinical diseases and play a critical role in pathogenesis (Pfau et al., 2011; Ludwig et al., 2017). Amphibole asbestos and silica exposure increases the number of autoantibodies targeting nuclear components (Pfau et al., 2005; Pfau et al., 2008; Foster et al., 2019) and alveolar macrophages during early apoptosis (Pfau et al., 2004; Blake et al., 2008). These autoantibodies are produced under homeostatic conditions by B-1 cells to remove dead and dying cells. Therefore, their elevated production likely results from the specific activation of B-1 cells in response to cytotoxic particles.

Indeed, in the early response to asbestos, peritoneal B-1 cells (and not B-2 cells) transiently lose alpha4 integrin-mediated adhesion, leading to their egress (Pfau et al., 2014a; Pfau et al., 2014b). B-1 cells migrate and accumulate in the injured zone and/or lymphoid organs (Ferro et al., 2014; Hiéronimus et al., 2021). The elevated proportion of B-1 cells in these organs persists for up to 8 months after exposure (Pfau et al., 2014a; Pfau et al., 2014b). Mechanistically, particles do not directly activate antibody production by CH12.LX B-1 cells. However, asbestos-exposed macrophages rapidly enhances spontaneous IgA and IgG production by CH12.LX B-1 cells (Rasmussen and Pfau, 2012). Similarly, peritoneal cells (rich in macrophages and B-1 cells (Ghosn et al., 2010)) exposed to asbestos *ex vivo* contained more IgA^+^ B-1 cells than those without exposure (Rasmussen and Pfau, 2012). The exact contribution of B-1 cells to the development or progression of particle-induced autoimmune disease needs to be investigated in more detail.

## 6 Summary

B-1 cells are mainly generated during fetal life and protect newborns against pathogens. B-1 cells are malleable and perform homeostatic functions, such as phagocytosis, natural antibody production, and IL-10 release. The pool of B-1 cells changes throughout life and even more after infection and exposure to chemicals/inorganic particles. B-1 cells can become autoantibody-producing cells (i.e., B-1 cell-derived plasma cells) or trigger granulomatous fibrosis (i.e., B1CDP), inflammation (i.e., IRA cells), or immunosuppression (i.e., B10). Possible ways to manipulate their functions to prevent the development of chemically induced or exacerbated pathologies remain unanswered. Finally, given the crucial roles of B-1 cells in this plethora of immune responses, they should not be overlooked when developing new *in vitro* models for predicting the toxicity of chemical compounds.

## Abbreviations

BCR, B cell receptor; TCR, T cell receptor; TLR, Toll-like receptor; AHR, Aryl hydrocarbon receptor; LPM, Large peritoneal macrophages; PC, Phosphorylcholine; MHC II, Class II major histocompatibility complex molecules; GM-CSF, Granulocyte-macrophage colony-stimulating factor; B1CD cell, B-1 cell-derived cell; B1CDP, B-1 cell-derived phagocytes; IRA, Innate response activator; RBC, Red blood cell; CS, Contact sensitivity; ED, Endocrine disruptors; HCB, Hexachlorobenzene; TCDD, 2,3,7,8-tetrachlorodibenzo-p-dioxin.
